# Recurrent Gastrointestinal Bleeding in an Elderly Patient With Peptic Ulcer Disease: Successful Management Through Multidisciplinary Intervention and Close Monitoring

**DOI:** 10.7759/cureus.41468

**Published:** 2023-07-06

**Authors:** Hamad Ahmad, Urooj Khan, Hoore Jannat, Noaman Ahmad

**Affiliations:** 1 Internal Medicine, Westchester Medical Center, Valhalla, USA; 2 Internal Medicine, Khyber Medical University, Peshawar, PAK; 3 Internal Medicine, Khyber Medical College, Peshawar, PAK; 4 Internal Medicine, Huntsville Hospital, Huntsville, USA

**Keywords:** transcatheter arterial embolization, multidisciplinary approach, refractory bleeding, recurrent bleeding, peptic ulcer disease, acute upper gastrointestinal bleeding

## Abstract

Upper gastrointestinal bleeding (UGIB) is a medical emergency with substantial morbidity and mortality worldwide. It is defined as bleeding originating in the gastrointestinal (GI) tract proximal to the ligament of Treitz and can be caused by various conditions, including peptic ulcers, gastritis, esophageal varices, Mallory-Weiss tears, and malignancies. Common complications include anemia, hemodynamic instability, perforation, and rebleeding. It is associated with high mortality and a poor prognosis, especially in high-risk populations. Management includes medical treatment, endoscopic intervention, endovascular arterial embolization, and surgery. We present an interesting case of an 87-year-old male with a history of duodenal ulcers who presented with a bleeding duodenal ulcer complicated by recurrent bleeding despite multiple hemostatic endoscopic interventions and arterial embolization. This case highlights the complexities involved in managing recurrent upper GI bleeding and emphasizes the significance of multidisciplinary approaches, timely interventions, and close monitoring.

## Introduction

Upper gastrointestinal bleeding (UGIB), a medical emergency with substantial morbidity and mortality, is defined as bleeding originating in the gastrointestinal (GI) tract proximal to the ligament of Treitz. Patients with acute UGIB commonly present with hematemesis and/or melena [1]. Hematochezia is usually due to lower GI bleeding; however, it can occur with massive upper GI bleeding [2]. Other associated symptoms, depending on etiology, include abdominal pain, dyspepsia, dysphagia, and odynophagia. Common causes of acute UGIB include peptic ulcer disease, esophageal varices, esophageal ulcers, Mallory Weiss tears, malignant ulcers, and malignancy [3]. Initial management depends on the patient’s hemodynamic status, though all patients should have at least two 18-gauge IV catheters and be nil per mouth. All patients with hemodynamic instability or active bleeding should be admitted to an intensive care unit for close observation, be adequately resuscitated with IV fluids, and have blood transfusions as indicated [4]. Adequate acid suppression with a proton pump inhibitor is of utmost importance and should be initiated as soon as the patient is optimally resuscitated [5]. Upper endoscopy is the diagnostic modality of choice for acute upper GI bleeding, with high sensitivity and specificity for locating and identifying bleeding lesions [6]. With recent advances, endoscopic procedures have been very successful in obtaining hemostasis; if unsuccessful or in cases of recurrent bleeding, endovascular arterial embolization is used [7]. We present an interesting case of an 87-year-old patient with a history of duodenal ulcers who presented with acute UGIB from a bleeding posterior duodenal ulcer refractory to endoscopic and endovascular arterial embolization.

## Case presentation

**Figure 1:**
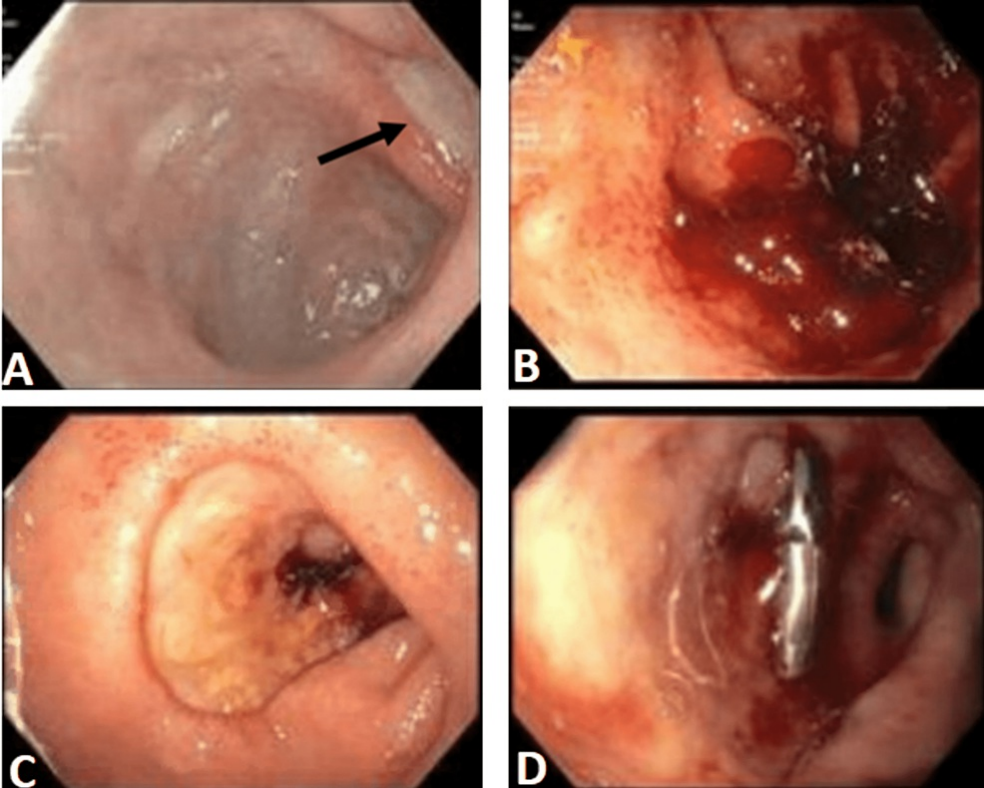
EGD showed clean base ulcer in the gastric antrum (A); Fresh blood in the gastric antrum (B); Visible bleeding vessel in the posterior duodenum (C); Endoclip deployed over the bleeding vessel (D) EGD: Esophagogastroduodenoscopy

An 87-year-old male with a past medical history of chronic back pain on ibuprofen, nonsteroidal anti-inflammatory drugs (NSAIDs)-induced duodenal ulcers, and hypertension was admitted for acute UGIB. The patient initially experienced epigastric pain, which he attributed to constipation and self-treated with enemas. However, a few days later, the patient noticed streaks of blood in the stools. Two weeks later, the patient was admitted for symptomatic anemia with a hemoglobin level of 8.6 mg/dl, down from 11 mg/dl at baseline. An esophagogastroduodenoscopy (EGD) revealed gastritis and a clean-based ulcer in the gastric antrum without active bleeding (Figure 1A). The patient was discharged to a subacute rehabilitation facility (SAR) with plans for a repeat EGD in three months and advised to avoid NSAIDs in the future.

One week later, the patient presented to the hospital again for symptomatic anemia. A complete blood count was significant for a low hemoglobin level of 7.3 mg/dl on arrival that dropped to 4.8 mg/dl the next day. A complete metabolic panel was essentially normal. The patient was admitted to the ICU and received multiple packed red blood cell transfusions, resulting in an improvement in hemoglobin to 9.1 mg/dl. The patient underwent a repeat EGD, revealing a gastric antrum filled with fresh blood (Figure 1B). Three endoclips were deployed, resulting in successful hemostasis. The patient's hemoglobin dropped to 6.6 the following day, along with hypotension (blood pressure (BP) 80/50 mmHg). The patient received packed red blood cells (pRBCs) and intravenous fluid boluses. The patient underwent emergent EGD and was found to have continuous ooze from the posterior duodenal bulb ulcer (Figure 1C). Multiple attempts could not result in hemostasis with endoclips (Figure 1D) and hemosprays.

**Figure 2:**
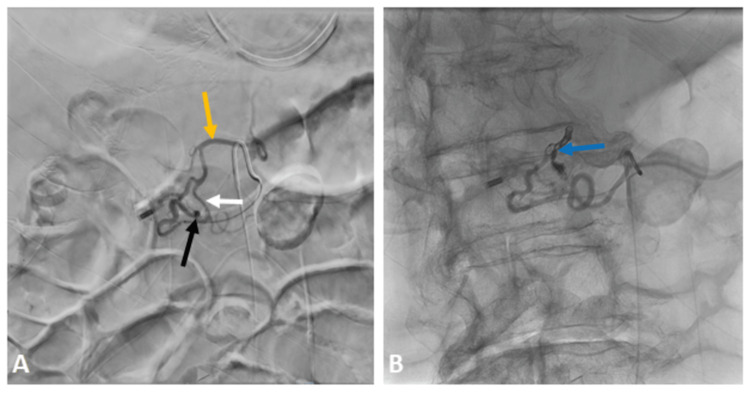
A: Angiography showed ooze from the gastroduodenal artery (black arrow); Gastroduodenal artery (white arrow) is a branch of the common hepatic artery (yellow arrow); B: Gastroduodenal artery embolization with gel and coil (blue arrow)

The patient was taken for emergent arterial embolization by interventional radiology. Angiography showed active spurting from the gastroduodenal artery (GDA) (Figure 2A), and the patient underwent successful gastroduodenal artery embolization (Figure 2B). The patient was transferred to the general floor.

Two days later, the nurse found the patient to have a bright red, bloody bowel movement accompanied by hypotension with systolic readings around 80 mmHg and a resultant drop in hemoglobin levels from 9 mg/dl to 6.8 mg/dl. The patient received pRBCs and fluid boluses and was transferred to the ICU for closer monitoring. A repeat emergent endoscopy showed active spurting in the posterior duodenal bulb. Epinephrine was injected at the base circumferentially, which decreased bleeding. A single endoclip was deployed to the vessel, resulting in successful hemostasis (Figure 1D). The patient stayed in the ICU for two days and was later transferred to the floor. The patient continued to remain hemodynamically stable, with no melena or hematochezia or a significant drop in hemoglobin. 

## Discussion

Endoscopic therapy is indicated for the treatment of most ulcers with stigmata of recent hemorrhage that increase the risk of recurrent bleeding. Katchinski et al. report that even after appropriate treatment, high-risk lesions with the presence of blood, clots, or bleeding vessels have a 5-20% rate of recurrent bleeding. However, low-risk lesions with a clean base or a flat pigmented spot have a relatively low rate of recurrent bleeding (3-10%) [8]. In the present case, the first EGD on previous admission showed duodenal ulcers but without any stigmata of recent bleeding, such as the presence of old blood, clots, or an actively bleeding vessel. Hence, the patient was treated with a proton pump inhibitor without any endoscopic intervention. However, a second endoscopy a week later showed an active bleeding vessel, which is associated with a high risk of rebleeding and mortality and was clipped endoscopically. Two days later, a third emergent endoscopy for an acute drop in hemoglobin showed a similar picture with an active rebleed not amenable to endoscopic intervention. Qvist et al. reported that in spite of medical management and/or endoscopic intervention, severe rebleeding can occur in 5% of patients, and such patients require surgery or transcatheter arterial embolization (TAE) [9]. Since surgery after failed endoscopic intervention is associated with a mortality rate as high as 40% [10], for high-risk patients (elderly and with comorbidities), TAE is a more favorable alternative [11]. Also, Rösch et al. reported more than 40 years ago that the use of TAE for the management of acute UGIB has increased [12]. Loffroy et al. evaluated the efficacy and medium-term outcomes of TAE for gastroduodenal ulcer bleeding after failed endoscopic intervention in high-risk patients and found that it resulted in 100% bleed control but was associated with an 18% rebleed rate [13]. The studied case underwent emergent gastroduodenal artery embolization, which resulted in successful hemostasis. However, two days later, the patient had a rebleed with hemodynamic decompensation. Kyaw et al. reported that one of the reasons for rebleeding post-embolization is due to the dual supply of the GDA from both the hepatic artery and the superior mesenteric artery. Hence, coiling the gastroduodenal artery solely could result in continued bleeding or the risk of recurrent bleeding from the superior mesenteric artery [14]. Since our patient had only the GDA embolization, the likely source of rebleeding was the superior mesenteric artery. The patient emergently underwent a fourth endoscopy with resultant hemostasis by endoclip deployment. The patient remained stable with no further episodes of melena or hemodynamic decompensation. Patient was discharged with close follow-up with GI.

## Conclusions

This case underscores the complexities encountered in the management of acute UGIB associated with peptic ulcer disease, particularly in elderly individuals. Even in cases where the bleeding source is not identified during endoscopy, it is crucial to exercise vigilant monitoring in high-risk patients. Should the condition progress to recurrent or refractory bleeding, a multidisciplinary approach becomes imperative. While TAE demonstrates a considerable success rate in achieving hemostasis in GI bleeding, the occurrence of rebleeding poses a significant threat to life, necessitating diligent surveillance and close observation.

## References

[1] Cappell MS, Friedel D (2008). Initial management of acute upper gastrointestinal bleeding: from initial evaluation up to gastrointestinal endoscopy. Med Clin North Am.

[2] Jensen DM, Machicado GA (1988). Diagnosis and treatment of severe hematochezia. The role of urgent colonoscopy after purge. Gastroenterology.

[3] Laine L, Barkun AN, Saltzman JR, Martel M, Leontiadis GI (2021). ACG clinical guideline: upper gastrointestinal and ulcer bleeding. Am J Gastroenterol.

[4] Baradarian R, Ramdhaney S, Chapalamadugu R (2004). Early intensive resuscitation of patients with upper gastrointestinal bleeding decreases mortality. Am J Gastroenterol.

[5] Leontiadis GI, Sharma VK, Howden CW (2005). Systematic review and meta-analysis of proton pump inhibitor therapy in peptic ulcer bleeding. BMJ.

[6] Jutabha R, Jensen DM (1996). Management of upper gastrointestinal bleeding in the patient with chronic liver disease. Med Clin North Am.

[7] Loffroy R, Guiu B, D'Athis P (2009). Arterial embolotherapy for endoscopically unmanageable acute gastroduodenal hemorrhage: predictors of early rebleeding. Clin Gastroenterol Hepatol.

[8] Katschinski B, Logan R, Davies J, Faulkner G, Pearson J, Langman M (1994). Prognostic factors in upper gastrointestinal bleeding. Dig Dis Sci.

[9] Qvist P, Arnesen KE, Jacobsen CD, Rosseland AR (1994). Endoscopic treatment and restrictive surgical policy in the management of peptic ulcer bleeding. Five years' experience in a central hospital. Scand J Gastroenterol.

[10] Lau JY, Sung JJ, Lam YH (1999). Endoscopic retreatment compared with surgery in patients with recurrent bleeding after initial endoscopic control of bleeding ulcers. N Engl J Med.

[11] Barkun AN, Bardou M, Kuipers EJ, Sung J, Hunt RH, Martel M, Sinclair P (2010). International consensus recommendations on the management of patients with nonvariceal upper gastrointestinal bleeding. Ann Intern Med.

[12] Rösch J, Dotter CT, Brown MJ (1972). Selective arterial embolization. A new method for control of acute gastrointestinal bleeding. Radiology.

[13] Loffroy R, Guiu B, Cercueil JP (2008). Refractory bleeding from gastroduodenal ulcers: arterial embolization in high-operative-risk patients. J Clin Gastroenterol.

[14] Kyaw M, Tse Y, Ang D, Ang TL, Lau J (2014). Embolization versus surgery for peptic ulcer bleeding after failed endoscopic hemostasis: a meta-analysis. Endosc Int Open.

